# Electric-Field-Assisted Co-Deposition of Bacteriorhodopsin and PEDOT:PSS on Interdigitated Electrodes for Biohybrid Photodetectors

**DOI:** 10.3390/bios16070398

**Published:** 2026-07-22

**Authors:** Abraham Ruiz Gómez, Juan Carlos Ferrer Millán, José Luis Alonso Serrano, Alba Hortal Foronda, Susana Fernández de Ávila López

**Affiliations:** 1 Communications Engineering Department, Miguel Hernandez University, 03202 Elche, Spain; 2 University Institute for Engineering Research, Miguel Hernandez University, 03202 Elche, Spain

**Keywords:** bacteriorhodopsin, PEDOT:PSS, biohybrid photodetectors, interdigitated electrodes, electric-field-assisted deposition, bioelectronics

## Abstract

This work presents the fabrication and characterization of biohybrid optoelectronic devices based on the integration of bacteriorhodopsin (bR) and PEDOT:PSS on interdigitated electrodes (IDEs). A three-phase methodology was developed to systematically optimize the active layer. First, the effect of an electric field applied during PEDOT:PSS drying was investigated, identifying a drying voltage of 1.2 V as the optimum among the tested conditions, achieving a maximum responsivity of (35.65±1.36) mA/W, a minimum noise equivalent power of $\left(5.99\pm 0.13\right)\times 10^{-10}$ W·H$z^{-1/2}$, and a maximum specific detectivity of $\left(3.55\pm 0.07\right)\times 10^{8}$ cm H$z^{1/2}$$W^{-1}$. Second, the compatibility of a physiological buffer for bR stabilization was assessed, demonstrating that a 60% PEDOT:PSS/40% buffer composition preserved and further improved the optoelectronic performance of the polymer matrix. Finally, bacteriorhodopsin was incorporated into the optimized PEDOT:PSS/buffer formulation, yielding the hybrid bR_1.2V_60% device, which exhibited an on/off ratio of 2.45 at 0 V and a maximum responsivity of (5.63±0.76) mA/W under reverse bias. Morphological analysis suggested improved film homogeneity, together with a continuous polymer matrix containing dispersed crystalline buffer-salt inclusions. Dynamic measurements showed a stable and reversible short-term photoresponse over successive illumination cycles ($\Delta I_{\mathrm{ON}}=18.54\pm 0.17$ μA) with full baseline recovery. These results demonstrate a scalable strategy for the development of biohybrid photodetectors with potential for low-power and sustainable optoelectronic applications.

## 1. Introduction

Currently, the development of novel sustainable materials and optoelectronic devices represents a key challenge in response to increasing technological demands [1] and the urgent need to mitigate environmental impact [2]. In this context, systems based on functional proteins, such as bacteriorhodopsin (bR), have emerged as promising alternatives to conventional synthetic materials [3]. Bacteriorhodopsin is a light-sensitive protein [4] located in the cell membrane of *Halobacterium salinarum* [5], functioning as a visible-light-activated proton pump that generates electrochemical potentials with high quantum efficiency [6].

**Figure 1:**
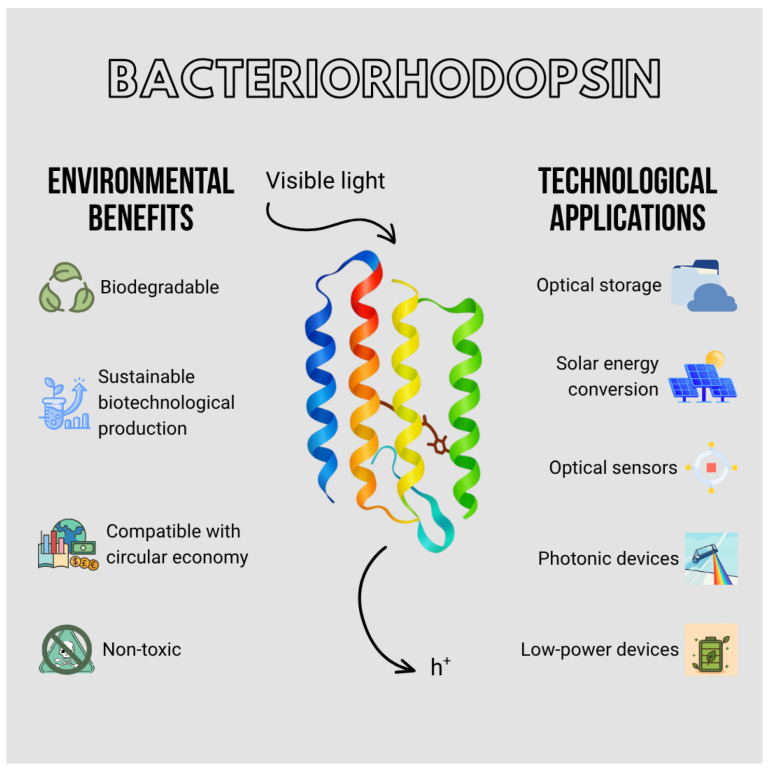
Schematic representation of bacteriorhodopsin as a sustainable biooptoelectronic material. The protein, activated by visible light, functions as a proton pump (H^+^), enabling applications in optical storage, solar energy conversion, sensors, photonic, and low-power devices. Its biocompatibility, biodegradability, and production through sustainable biotechnological methods highlight its environmental advantages over conventional synthetic materials.

The optoelectronic properties of bacteriorhodopsin—including its thermal stability [7] and reversible photochromic behavior [8]—position it as an ideal biofunctional material for applications in photonics [9], optical data storage [10], sensors [11], and solar energy conversion devices [12], as schematically illustrated in Figure 1. Unlike traditional inorganic semiconductors, this protein can be produced through sustainable biotechnological cultivation [13], is biodegradable [14], and operates without the need for extreme conditions [15] or toxic materials [16]. Moreover, its integration into hybrid platforms and solid-state devices has demonstrated feasibility in the fabrication of low-power optoelectronic systems [17], providing an eco-friendly alternative grounded in principles of biofabrication [16]. Thus, the utilization of natural proteins such as bacteriorhodopsin not only constitutes a significant advancement in the field of biooptoelectronics but also offers a pathway for the development of clean [18], renewable [19], and multi-functional technologies [20] aligned with sustainability goals and the principles of the circular economy within materials science and applied engineering.

### 1.1. Poly(3,4-ethylenedioxythiophene):poly(styrene sulfonate), PEDOT:PSS

**Figure 2:**
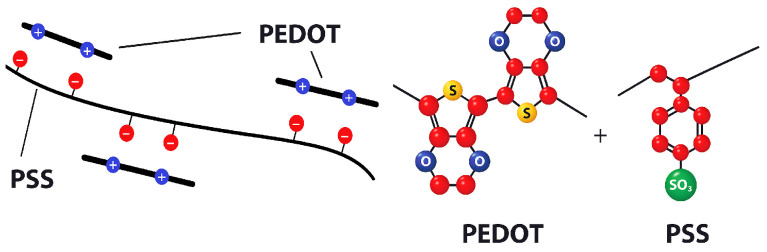
Schematic representation of the PEDOT:PSS complex, showing the electrostatic interaction between PEDOT (blue, conductive polymer) and PSS (red, polyelectrolyte matrix). Molecular structures of PEDOT and PSS are also depicted, illustrating their complementary roles in conductivity and solubility.

Within the framework of the search for sustainable and biofunctional optoelectronic materials, poly(3,4-ethylenedioxythiophene):poly(styrene sulfonate), commonly known as PEDOT:PSS (Figure 2), has established itself as one of the most relevant conducting polymers for advanced applications [21]. This complex copolymer combines the electronic properties of PEDOT with the solubility and processability of PSS [22,23,24], enabling its use in solution-based deposition techniques such as spin-coating [25], inkjet printing [26], and screen printing [27]. These characteristics facilitate its integration into devices fabricated on low-cost [28], flexible substrates [29].

One of the most remarkable properties of PEDOT:PSS is its electrical conductivity [30], which can be tuned from moderate values to levels comparable to those of certain doped semiconductors through post-deposition treatments with polar solvents such as dimethyl sulfoxide (DMSO) [31] or strong acids [32]. This tunability makes the material an excellent candidate for transparent electrodes [33], electronic interfaces [34], and active layers in optoelectronic devices [35]. Additionally, its high optical transparency in the visible range is advantageous for photonic applications [36], including solar cells [37], organic light-emitting diodes [36], and optical sensors [38].

Moreover, PEDOT:PSS exhibits remarkable mechanical flexibility, offering a clear advantage over brittle inorganic materials such as indium tin oxide (ITO). This flexibility enables its use in portable, wearable, and implantable electronic systems [39]. Such a property is particularly valuable for the development of biointeractive platforms and biohybrid structures, where the material must retain electronic functionality under repeated mechanical deformation.

A critical property for its integration into bio-optoelectronic systems is its biocompatibility [40,41]. Numerous studies have demonstrated that PEDOT:PSS supports cell growth [42,43,44], interacts favorably with biological tissues [45], and functions effectively as an interface in biosensors [46] and neuromorphic devices [47]. This attribute, combined with its ability to conduct both ionic and electronic charges (mixed conduction), makes it a highly efficient mediator for coupling living systems with electronic circuits.

In this context, PEDOT:PSS serves as an ideal bridging material between organic and inorganic domains, facilitating the incorporation of biological components [48], such as photoactive proteins, into hybrid optoelectronic architectures. Its application alongside functional biomolecules enables the development of advanced, sustainable, and bioinspired devices, aligned with emerging technological strategies aimed at achieving greener, more adaptable, and biologically compatible electronics [49]. In this context, recent paradigms in organic mixed ionic–electronic conductors have highlighted these blended systems as foundational tools for tailoring operation mechanisms in advanced bioelectronic sensors [50].

### 1.2. Towards the Integration of Bacteriorhodopsin into PEDOT:PSS Matrices

The convergence of conductive organic materials with functional proteins represents an emerging strategy for the development of hybrid bio-optoelectronic devices [51]. Within this framework, bacteriorhodopsin and PEDOT:PSS exhibit highly complementary properties that enable the design of integrated platforms with advanced functionality and enhanced sustainability [52]. While PEDOT:PSS provides a conductive, flexible, and biocompatible matrix, bacteriorhodopsin acts as a photoactive element capable of transforming light energy into electrochemical signals [53]. Furthermore, recent advances have extended its versatility into alternative bio-interfaces, including its adaptation in solid-state pH sensors for monitoring biological environments [54]. Integrating these two components into a single functional architecture is not only conceptually compelling but also technically feasible, as demonstrated by growing experimental evidence in the field of bio-sensitized solar cells [55] and bioelectronic systems [56].

In this context, the fabrication of hybrid devices based on bacteriorhodopsin requires precise control over the orientation and immobilization of the protein in order to ensure the generation of a directional photoelectric response of sufficient magnitude [57]. Furthermore, it is essential to preserve the structural integrity of the purple membrane (PM), as its denaturation would lead to the irreversible loss of photoactive function [58]. Over the past decades, numerous methods have been proposed for the deposition and alignment of bR layers onto various substrates; among them, the electrophoretic sedimentation (ES) technique has been demonstrated as an effective approach for the fabrication of oriented bacteriorhodopsin films, enabling controlled alignment and measurable electrochemical/photoelectric response [59]. In particular, ES stands out as a practical and low-cost technique capable of inducing partial dipolar alignment of bR under an applied electric field, making it especially attractive for integration with solution-processed conductive polymers such as PEDOT:PSS in flexible biohybrid devices.

The extrapolation of these strategies to PEDOT:PSS systems presents both challenges and opportunities. On one hand, the hydrophilic nature of PEDOT:PSS, together with its abundance of sulfonate functional groups, can promote the immobilization of bacteriorhodopsin through controlled electrostatic and hydrophobic interactions [60]. Nevertheless, although this approach offers an improvement over random adsorption, the achieved molecular orientation is not always completely uniform. As a result, a fraction of bR molecules may adopt an opposite alignment, which can interfere with proton transport processes and consequently reduce the efficiency of the photoelectric response [61]. The ES technique—previously shown to enhance protein alignment and photoresponse—could be adapted for thin PEDOT:PSS films deposited on conductive substrates. Since PEDOT:PSS can be processed from solution and applied to flexible supports, the application of an external electric field during immobilization may promote a controlled dipolar orientation of bR, thereby maximizing its functional efficiency within a biohybrid device [58,61].

### 1.3. Electric-Field-Assisted Co-Deposition of Bacteriorhodopsin and PEDOT:PSS for Enhanced Biohybrid Device Fabrication

A particularly attractive strategy for achieving the functional integration of bacteriorhodopsin into PEDOT:PSS matrices involves the co-deposition of both components, followed by an immobilization process assisted by an electric field during the drying stage. In this approach, a homogeneous mixture of PEDOT:PSS and bacteriorhodopsin in aqueous solution can be applied onto a conductive substrate using drop-casting techniques. During solvent evaporation, the application of an external electric field can induce partial directional alignment of polar species according to their intrinsic dipole moments, a phenomenon reported in conducting polymers such as PEDOT:PSS, where electric-field-induced dipolar reorientation leads to measurable changes in morphology and anisotropic electrical properties.

Although the use of electric-field-assisted alignment has not been extensively explored in PEDOT:PSS–bR composite systems, previous studies have demonstrated that bacteriorhodopsin is sensitive to external electric fields, which can modulate its photoelectric response and proton transport behavior, particularly in oriented films [57]. Furthermore, the functional performance of bR-based systems is strongly dependent on molecular orientation, as the directionality of proton pumping is inherently linked to the spatial arrangement of its transmembrane domains [11]. The PEDOT:PSS polymer matrix not only provides mechanical flexibility and electronic conductivity, but may also contribute to the stabilization of embedded biomolecules by offering a hydrated and ionically active environment, as reported for bioelectronic interfaces based on conducting polymers [48,62]. However, the extent to which this environment mitigates protein denaturation or aggregation during solvent evaporation remains dependent on the specific system and processing conditions. These features are particularly relevant for applications in photodetectors, biosensors, and bio-optoelectronic devices, where interfacial organization and charge density play a central role.

### 1.4. Deposition on Interdigitated Electrodes for Biohybrid Devices

Interdigitated electrodes (IDEs) constitute a key architecture for PEDOT:PSS-based platforms, particularly in biohybrid and sensing devices [63]. Their interlocking finger design provides a large active surface area and a well-defined electric field distribution, promoting efficient interaction between the conductive layer and active materials. The deposition of PEDOT:PSS and biofunctional components onto IDEs via solution-based techniques such as drop-casting or printing represents an effective strategy for developing high-performance bioelectronic devices [48]. In the biomedical field, IDE-based sensors functionalized with PEDOT:PSS and biomolecules enable highly sensitive detection through variations in conductivity, impedance, or photoelectrical response [64]. These systems can identify ions, biomolecules, or pathogenic agents, offering promising solutions for clinical diagnostics and environmental monitoring. Moreover, bacteriorhodopsin’s intrinsic ability to convert light into electrical signals has been widely explored in bio-optoelectronic devices, enabling light-driven sensing and energy transduction [11]. Another prominent application is the development of wearable and implantable devices for continuous physiological monitoring, where PEDOT:PSS plays a central role due to its flexibility, transparency, and mixed ionic–electronic conductivity [39]. The integration of photoactive proteins such as bacteriorhodopsin further expands the functionality of these systems toward self-powered sensing platforms.

In the context of cutting-edge biomedical paradigms, the convergence of light-sensitive proton pumps with flexible, mixed ionic–electronic conductors on interdigitated microstructures addresses critical milestones in neuroprosthetics and clinical interfaces. Specifically, biohybrid photodetectors based on bacteriorhodopsin and PEDOT:PSS offer a biomimetic foundation for the development of artificial retinas and subretinal visual prostheses capable of light-driven cellular stimulation without requiring external, bulky power units [65]. Furthermore, the intrinsic mechanical flexibility and high biocompatibility of these organic matrices overcome the severe elastic mismatch and subsequent chronic immune rejection typical of conventional rigid silicon-based implants, thereby enabling stable, long-term biointeractive optoelectronic interfaces for real-time physiological and metabolic tracking [66]. From a broader societal perspective, transitioning toward biofabrication methodologies that utilize non-toxic, biodegradable proteins and polymers directly counters the escalating global crisis of healthcare-associated electronic waste generated by single-use diagnostic hardware. Consequently, advancing this biohybrid platform provides society with minimally invasive, highly compatible therapeutic tools and green point-of-care diagnostics, tightly aligning immediate clinical demands with global eco-sustainability goals [67].

The objective of the present work is to develop a methodology for the simultaneous deposition of PEDOT:PSS and bacteriorhodopsin onto interdigitated electrodes using a solution-based coating technique assisted by an electric field. This approach aims to immobilize the protein within the polymer matrix by exploiting its intrinsic dipole moment, promoting partial alignment during the drying process and enhancing the functional efficiency of the biohybrid system. To the best of our knowledge, no prior studies have reported the combined use of co-deposition and electric-field-assisted orientation of bacteriorhodopsin within PEDOT:PSS matrices on IDE architectures, highlighting the novelty of this approach.

## 2. Materials and Methods

### 2.1. Materials

An aqueous dispersion of PEDOT:PSS (Clevios, PH1000, 1.0–1.3 wt% in water) was used as the conductive matrix. This material exhibits high optical transparency in the visible range, mechanical flexibility, and good electrical conductivity, making it suitable for integration into hybrid and electronic devices. The material was used as received, without additional purification or modification steps.

The bacteriorhodopsin protein, provided for this work by the R&D department of Bras del Port S.A. (Santa Pola, Spain), was obtained from previously isolated cultures of *Halobacterium salinarum*. The sample consisted of lyophilized purple membrane vesicles, which exhibit high structural stability and preserved photoelectrical response. This format facilitates rehydration and subsequent incorporation into aqueous solutions for optoelectronic applications. As with PEDOT:PSS, the protein was used without further purification in order to maintain experimental conditions compatible with scalable and sustainable fabrication processes.

**Figure 3:**
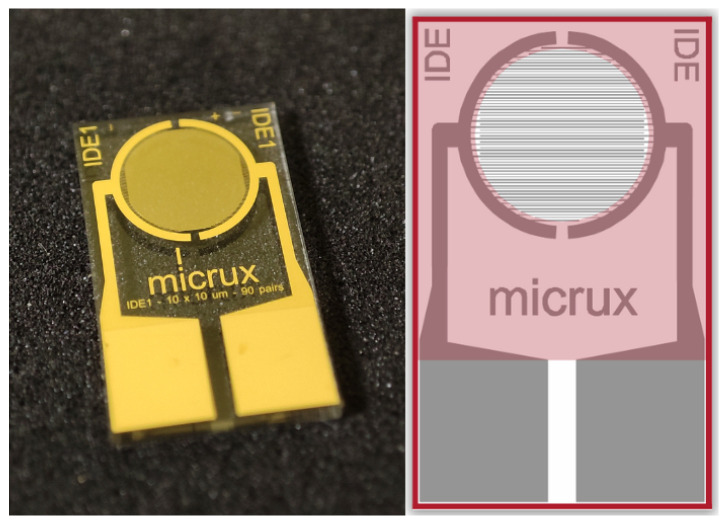
The interdigitated electrode used in this study from Micrux Technologies.

The interdigitated electrode (IDE) used in this study was purchased from Micrux Technologies (Figure 3). The device is fabricated on a glass substrate with external dimensions of 10 × 6 × 0.7 mm and features gold (Au) interdigitated electrodes with a width and spacing of 10 μm. The electrode structure consists of a bilayer with 50 nm of titanium (adhesion layer) and 150 nm of gold (conductive layer). This configuration provides a well-defined electric field distribution and a high surface-to-volume ratio, enabling efficient interaction between the active material and the electrodes.

The total length of conductive gold within the IDE was estimated based on the number of interdigitated fingers in the active sensing area. Considering a pitch of 20 μm (10 μm electrode width and 10 μm spacing) and a sensing diameter of 3 mm, approximately 150 fingers were obtained. Assuming each finger spans the full diameter, the total gold length is approximately 450 mm (45 cm). With a line width of 10 μm (0.01 mm), the total gold-covered area is estimated to be 0.045 cm^2^.

### 2.2. Methods

For the preparation of the active blend, bacteriorhodopsin (bR) was first dispersed in a pH 7.4 buffer solution in order to ensure structural stability and preserve its photoelectric functionality under near-physiological conditions. The buffer consisted of 10 mM Tris-HCl and 100 mM NaCl, with the pH adjusted to 7.4 using 1 M HCl. It was prepared by dissolving 1.21 g of Tris base (molar mass: 121.14 g/mol) and 5.84 g of NaCl (molar mass: 58.44 g/mol) in ultrapure water (Milli-Q), followed by adjustment of the final volume to 1000 mL. The solution was gently stirred until complete dissolution was achieved.

The bR dispersion was subsequently mixed with the aqueous PEDOT:PSS solution in predefined ratios depending on the experimental series under study. For the voltage optimization experiments, PEDOT:PSS films were dried under different applied voltages while keeping the rest of the deposition conditions constant. For the buffer optimization experiments, PEDOT:PSS and buffer solution were mixed in the volumetric proportions defined for each formulation. Based on the optimal drying voltage obtained from the first experimental series and the optimal PEDOT:PSS/buffer composition determined in the second series, the hybrid bacteriorhodopsin/PEDOT:PSS devices were then prepared using the selected PEDOT:PSS/buffer composition and drying voltage. In all cases, the resulting mixture served as the precursor solution for the functional layer to be deposited onto the interdigitated electrodes.

Thin films were fabricated by drop-casting, selected for its simplicity, reproducibility, and compatibility with solution-based processing. In each case, a fixed volume of 5 μL was deposited onto the active area of the interdigitated electrode. The deposition was performed after the electrical bias had already been applied to the IDE, and the applied voltage was maintained constant throughout the entire drying process. The droplet spread over the substrate due to surface tension and wetting effects, forming a liquid film that covered the active area. Since the deposited volume was kept constant in all experiments, the main variables governing film formation were the composition of the precursor solution and the electric field applied during drying.

During the drying stage, the sample was subjected to a controlled electric field applied parallel to the plane of the substrate through the interdigitated electrode geometry. The voltage applied during drying was defined according to each experimental series and maintained constant until complete solvent evaporation. This procedure was intended to promote molecular organization within the deposited layer and, in the case of the hybrid films, to potentially influence the spatial distribution of bacteriorhodopsin within the PEDOT:PSS matrix. Drying was carried out at room temperature under controlled humidity conditions. Once dried, the devices from each experimental series were placed in a vacuum chamber, where a pressure of $10^{-3}$ Torr was applied prior to electrical and optoelectronic characterization. This step was performed to minimize the influence of residual moisture and atmospheric adsorbates on the electrical response of the devices.

The nomenclature of the devices was established according to the fabrication conditions used in each case. In the first experimental series, PEDOT:PSS reference devices were identified by the voltage applied during drying (OPD_0.0 V, OPD_0.6 V, OPD_1.2 V, and OPD_1.8 V). In the second series, control devices containing PEDOT:PSS and buffer solution were labeled according to the volumetric percentage of PEDOT:PSS in the precursor mixture (OPD_20%, OPD_40%, OPD_60%, and OPD_80%). Finally, the hybrid devices containing bacteriorhodopsin were labeled according to the optimized drying voltage and PEDOT:PSS/buffer composition used for their preparation.

Electrical measurements were performed using a Keithley 2400 SourceMeter controlled through a LabVIEW application via a General Purpose Interface Bus (GPIB). Illumination measurements were performed under simulated 1 sun illumination (AM1.5 G) using a Newport solar simulator equipped with a 150 W xenon lamp as the light source. After complete drying, the devices were subjected to optoelectronic characterization under dark and illuminated conditions in order to evaluate their functional response.

To quantitatively evaluate the optoelectronic performance and establish a physically rigorous framework for comparing the biohybrid device with the state-of-the-art literature, the figures of merit were calculated using standardized formulations [68]. The calculations incorporate the incident light power density of the illumination source ($P_{in}$) and the geometrically calibrated effective active area of the interdigitated electrode channels ($A=0.045\;{\mathrm{cm}}^{2}$), derived from the 3mm effective sensing diameter. The Responsivity (*R*, in $A\cdot W^{-1}$ or $\mathrm{mA}\cdot W^{-1}$) was determined via Equation (1):

(1) $$R=\frac{I_{light}-I_{dark}}{P_{in}\cdot A}$$

Inorganic mixed ionic–electronic conductors (OMIECs) structured on interdigitated layouts, estimating the noise floor exclusively from the dark current magnitude often leads to severe underestimations. The finite inherent conductivity of the film necessitates the inclusion of thermal carrier fluctuations. Therefore, the total noise current density ($I_{n}$, in $A\cdot {\mathrm{Hz}}^{-1/2}$) was mathematically defined as a comprehensive single-sided spectral density combining both shot noise and Johnson–Nyquist thermal noise components (Equation (2)) [68]:

(2) $$I_{n}=\sqrt{2qI_{dark}+\frac{4k_{B}T}{R_{sh}}}$$

where *q* is the elementary electronic charge ($1.602\times 10^{-19}\;C$), $k_{B}$ is the Boltzmann constant ($1.38\times 10^{-23}\;J/K$), *T* is the operational room temperature (298K), and $R_{sh}$ is the exact shunt resistance derived from the inverse slope of the dark I–V characteristics corresponding to each device configuration.

Consequently, the Noise Equivalent Power (NEP, in $W\cdot {\mathrm{Hz}}^{-1/2}$) and the Specific Detectivity ($D^{*}$, in Jones, $\mathrm{cm}\cdot {\mathrm{Hz}}^{1/2}\cdot W^{-1}$) were calculated strictly using Equations (3) and (4):

(3) $$NEP=\frac{I_{n}}{R}$$

(4) $$D^{*}=\frac{\sqrt{A\cdot \Delta f}}{NEP}$$

To prevent physical inconsistencies, the equivalent noise bandwidth was explicitly normalized to Δf=1Hz. By factoring the thermal noise contributions governed by $R_{sh}$ alongside the geometric parameters, this methodology ensures a rigorous baseline assessment of the device detection limits.

## 3. Results and Discussion

### 3.1. Analysis of PEDOT:PSS Films Under Applied Voltage During the Drying Process

**Figure 4:**
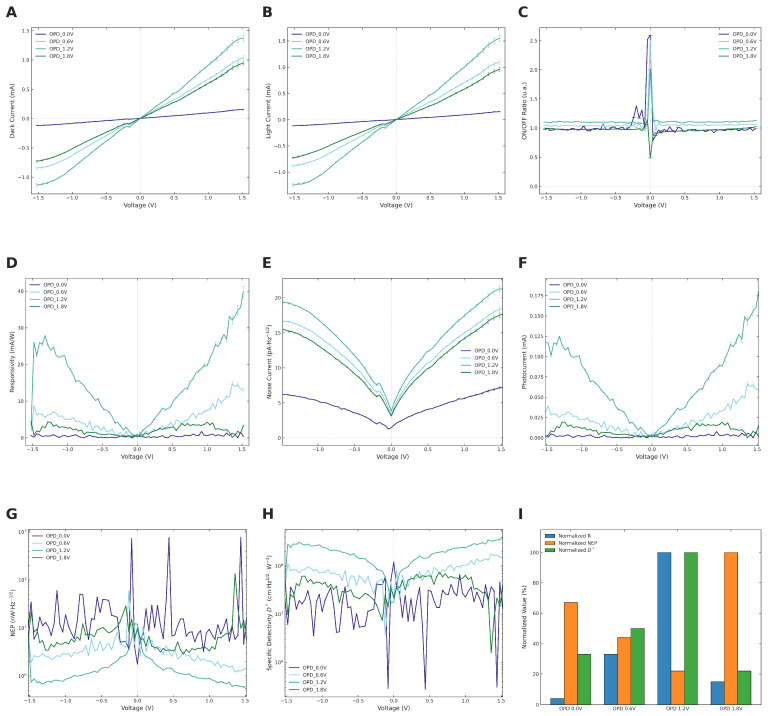
Optoelectronic characterization of PEDOT:PSS devices dried under different applied voltages. (**A**) Dark current–voltage curves. (**B**) Current–voltage characteristics under illumination. (**C**) On/off current ratio as a function of applied bias. (**D**) Responsivity curves. (**E**) Total noise current. (**F**) Net photocurrent. (**G**) Noise equivalent power. (**H**) Specific detectivity. (**I**) Bar chart comparing normalized performance parameters.

Figure 4 summarizes the electrical and optoelectronic performance of the OPD devices fabricated under different drying voltages. The dark and illuminated I–V characteristics (Figure 4A,B) exhibit a predominantly ohmic behavior over the entire polarization range, which is characteristic of PEDOT:PSS deposited on interdigitated electrodes, with no observable evidence of rectification. The most relevant effect of the drying voltage is reflected in the slope of the curves: OPD_1.2 V and OPD_0.6 V show the highest dark currents under reverse bias. We hypothesize that this could be associated with an increased macroscopic film conductivity, potentially driven by the reorientation of PEDOT chains under the applied electric field [69]. While macroscopic electrical improvements are clearly observed, it should be noted that molecular-level reorientation remains a proposed mechanism based on the literature rather than a directly verified structural fact in this study. This proposed mechanism is highly consistent with modern nanoarchitectonics frameworks elucidating the interplay between processing parameters and molecular orientation in polymer thin films [70].

Furthermore, we hypothesize that the pronounced performance drop observed at 1.8 V is intrinsically linked to the electrochemical window of the aqueous medium [71]. Since the PEDOT:PSS precursor is an aqueous dispersion, the theoretical thermodynamic limit for water electrolysis stands at 1.23 V [72]. Operating at 1.2 V keeps the system just below this threshold, likely allowing the maximum electric field strength for film processing without triggering massive solvent degradation. Conversely, increasing the bias to 1.8 V significantly exceeds this window. According to established electrochemical principles, this overpotential is expected to induce water splitting and the subsequent evolution of gas micro-bubbles (H_2_ and O_2_) during the delicate film-formation phase. The escape of these gases could generate structural defects, discontinuities, or micro-pinholes within the drying matrix [71,73]. Additionally, such excessive electrical bias may promote the irreversible electrochemical over-oxidation of PEDOT chains, which is a known degradation pathway that shortens conjugation lengths and ultimately impairs the overall conductivity of the device [74]. Under illumination, the separation between the curves corresponding to the different devices becomes more pronounced than in dark conditions, particularly under reverse bias. OPD_1.2 V exhibits the highest current under illumination in this regime, whereas OPD_0.0 V displays nearly overlapping dark and illuminated curves across the reverse bias range, suggesting that, in the absence of a drying field, charge carrier photogeneration under bias would be limited.

The on/off ratio (Figure 4C) directly reflects the light/dark discrimination capability of each device. Under reverse bias, OPD_1.2 V consistently exhibits the highest on/off ratio, reaching values of approximately 1.12, whereas OPD_0.0 V remains close to unity with noticeable dispersion. Responsivity (Figure 4D) is the parameter that shows the most pronounced differentiation among the devices. OPD_1.2 V consistently outperforms the other devices over the investigated voltage range, reaching a maximum responsivity of (35.65±1.36)mA/W (n=5) at V=+1.48 V, exceeding the value of OPD_0.0 V ((1.72±0.03) mA/W) by approximately 21 times, and those of OPD_1.8 V ((4.32±0.03) mA/W) and OPD_0.6 V ((14.82±0.54) mA/W) by factors of approximately 8 and 2.4, respectively. We propose that this non-monotonic dependence on drying voltage could indicate the existence of an optimal electric field strength for film processing, beyond which excessive voltage might introduce macroscopic structural disorder or over-oxidation in the film, although further structural studies would be required to fully elucidate this mechanism.

The total noise current (Figure 4E), estimated by considering both shot noise and Johnson thermal noise contributions through point-by-point differential resistance, exhibits a minimum close to V=0 V for all devices. The net photocurrent (Figure 4F) increases markedly for OPD_1.2 V under applied bias. Under reverse bias, this increase in photocurrent significantly outweighs the increase in noise, resulting in a higher signal-to-noise ratio. Consequently, the NEP (Figure 4G) decreases significantly as external bias is applied, reaching optimal detection thresholds; OPD_1.2 V attains a minimum of $\left(5.99\pm 0.13\right)\times 10^{-10}$ W·H$z^{-1/2}$ (n=5) at V=+1.48 V. Specific detectivity (Figure 4H) likewise reaches a maximum of $\left(3.55\pm 0.07\right)\times 10^{8}$ cm H$z^{1/2}$ $W^{-1}$ (n=5), establishing OPD_1.2 V as the most suitable configuration within the analyzed series. The normalized performance comparison shown in Figure 4I further confirms the balanced optimization achieved for OPD_1.2 V, which combines the highest normalized responsivity and detectivity with the lowest normalized NEP. The optimal values of the main figures of merit for each device within the investigated polarization range are summarized in Table 1.

### 3.2. Effect of Buffer Solution Concentration on the Optoelectronic Properties of PEDOT:PSS Films Dried at 1.2 V

Prior to the integration of bacteriorhodopsin into the active layer, the independent effect of the buffer solution required for protein stabilization was evaluated. A control experiment was conducted in which PEDOT:PSS was mixed with the buffer solution in varying volumetric proportions, ranging from 20% to 80% of the conductive polymer, while all resulting films were dried under the previously determined optimal applied voltage of 1.2 V. This approach enabled the assessment of the extent to which the buffer solution itself modifies the charge transport pathways and detection figures of merit before the introduction of the photoactive biological component. The volumetric compositions of PEDOT:PSS and buffer solution used to fabricate each control device are summarized in Table 2.

**Figure 5:**
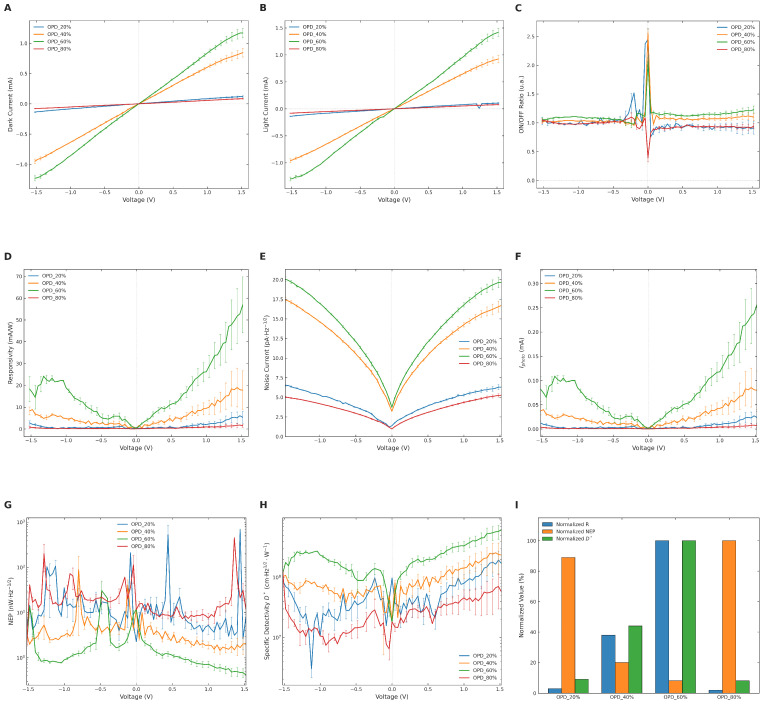
Electrical and optoelectronic characterization of control devices fabricated with different PEDOT:PSS/buffer ratios and dried at 1.2 V: (**A**,**B**) current–voltage (I–V) characteristics under different operating conditions, (**C**) on/off current ratio as a function of voltage, (**D**) responsivity, (**E**) noise current spectral density, (**F**) photocurrent, (**G**) noise equivalent power (NEP), (**H**) specific detectivity (D*) as a function of voltage, and (**I**) normalized comparison of responsivity (R), NEP, and D* for the different PEDOT:PSS/buffer ratios.

A clear trend is observed in the electrical response of the devices (Figure 5A,B): the conductivity progressively increases from OPD_20% to OPD_40%, reaching a maximum for the OPD_60% formulation. This behavior suggests that the incorporation of a buffer solution initially promotes improved charge transport within the PEDOT:PSS network, likely due to the ionic screening and structural reorganization effects commonly reported for PEDOT:PSS-based systems [75,76]. However, when the buffer proportion becomes excessive, as in the OPD_20% formulation, the conductive network becomes significantly disrupted, leading to poor electrical performance. In contrast, the OPD_80% device, despite containing a higher PEDOT:PSS fraction, exhibits a lower photoresponse than OPD_60%, suggesting that the intermediate composition provides a more favorable balance between conductivity and the overall optoelectronic response. Due to the solution-based drop-casting process onto the interdigitated electrode layouts, solvent evaporation naturally triggers a localized coffee-ring effect, which yields macroscopic thickness profile variations along the channel boundaries [77]. Consequently, standard decoupled characterizations—such as micro-domain Atomic Force Microscopy (AFM) or standalone four-point probe profiling—become highly prone to geometric artifacts and localized sampling variations, making them less representative of the global bulk properties. Instead, the net macroscopic optoelectronic metrics serve as a direct functional expression of the system’s configuration. We propose that this behavior is governed by the electronic–ionic transport trade-off characteristic of organic mixed ionic–electronic conductors (OMIECs) [50]. While the PEDOT:PSS network provides the necessary electronic pathways for charge extraction, the volume fraction of the physiological buffer sustains the hydration and ionic mobility required for biomolecular stability. Below 60% conductive polymer, the matrix approaches its electrical percolation threshold, restricting charge collection. Conversely, exceeding this ratio limits the aqueous microenvironment, suppressing efficient ionic coupling. Thus, the 60% composition appears to represent a functionally optimized operational balance rather than an absolute structural ideal. The OPD_60% device also maintains a higher and more stable on/off ratio throughout the usable voltage range (Figure 5C) and exhibits the highest responsivity (Figure 5D), reaching a maximum of (52.78±01.30) mA/W (n=5). Although the OPD_60% devices show higher noise levels than OPD_80% (Figure 5E), they simultaneously generate a substantially larger net photocurrent (Figure 5F). Consequently, the NEP and specific detectivity trends (Figure 5G,H), together with the normalized comparison shown in Figure 5I, identify OPD_60% as the best-performing formulation among the tested buffer concentrations.

The excellent photoelectric performance of OPD_60% demonstrates that the incorporation of a 40% buffer fraction does not significantly deteriorate the photoelectric properties of the PEDOT:PSS film while still providing the physicochemical conditions required for the stabilization of bacteriorhodopsin as the photoactive component of the active layer. This functional stability aligns with recent milestones demonstrating that, when managing contact parameters, proteins embedded in thin-film solid-state junctions can operate as highly efficient electronic conducting materials [78]. The corresponding figures of merit for the complete buffer concentration series are summarized in Table 3.

### 3.3. Optoelectronic Characterization of the Hybrid Bacteriorhodopsin/PEDOT:PSS Photodetector

**Figure 6:**
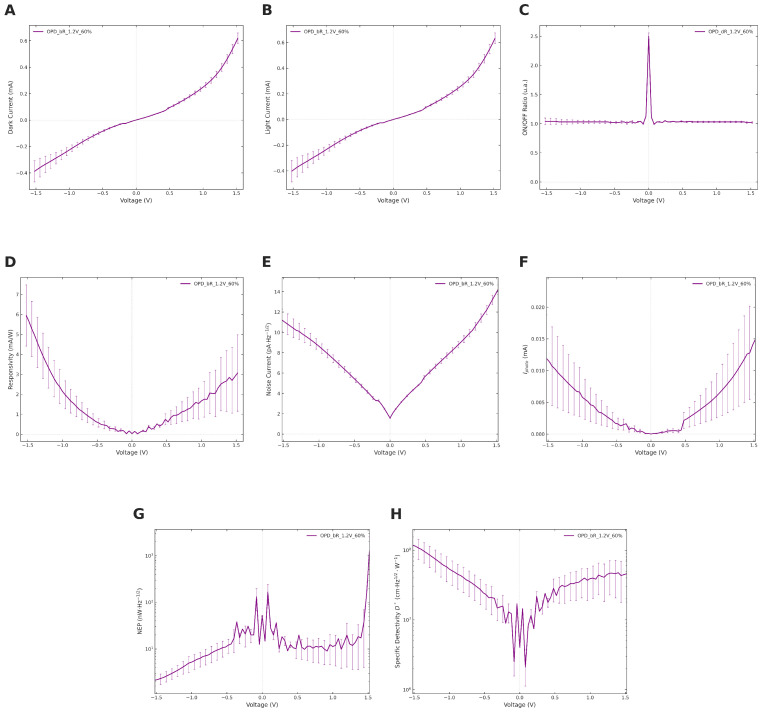
Optoelectronic characterization of the hybrid bR_1.2V_60% photodetector: (**A**,**B**) dark and illuminated current–voltage (I–V) characteristics, (**C**) on/off current ratio as a function of voltage, (**D**) responsivity, (**E**) noise current spectral density, (**F**) net photocurrent, (**G**) noise equivalent power (NEP), and (**H**) specific detectivity ($D^{*}$) as a function of applied voltage.

The current–voltage characteristics shown in Figure 6A,B exhibit a quasi-ohmic and nearly symmetric behavior around zero bias, although a moderate nonlinearity appears at higher applied voltages. In comparison with the PEDOT:PSS reference devices presented in Figure 5A,B, the hybrid bR_1.2V_60% device shows substantially lower dark current levels over the entire polarization range. In particular, the dark current at V=0 V is $8.66\times 10^{-8}$ A, approximately 15 times lower than that of the OPD_60% device without bacteriorhodopsin ($1.28\times 10^{-6}$ A), indicating that the incorporation of bacteriorhodopsin significantly alters the macroscopic charge transport dynamics within the polymer matrix. We hypothesize that this reduction in dark current may be associated with changes in the interfacial transport processes and carrier mobility within the hybrid film, contributing to the suppression of leakage currents under equilibrium conditions. Under illumination, the current–voltage curve clearly separates from the dark response in most of the applied bias range (Figure 6B), confirming the photoelectrical response of the hybrid device, which is commonly associated with the photoactive and charge-transfer properties of systems based on bacteriorhodopsin [3,6].

The on/off ratio shown in Figure 6C reaches a maximum value of 2.45 at V=0 V, demonstrating a measurable empirical photoresponse under zero-bias conditions. It should be noted that the on/off ratio rapidly approaches unity at higher applied biases; this is not due to a cessation of photoactivity, but rather because the massive bulk dark current of the conductive PEDOT:PSS matrix under bias computationally overwhelms the relatively small, stable photocurrent contribution of the biological component. This measurable photocurrent generated at zero bias strongly suggests the presence of an internal driving force. However, because the interdigitated electrodes utilized in this architecture are structurally symmetric (employing the same metal for both contacts), a macroscopic rectification ratio from the dark I–V curves was extracted to definitively prove this is unfeasible. Therefore, we propose a theoretical model wherein localized internal asymmetries or a built-in potential gradient within the complex hybrid structure enables spontaneous charge separation under illumination. This is presented strictly as a hypothesis, and specialized transient studies would be required to definitively map these internal fields.

The responsivity profile (Figure 6D) reaches a maximum value of 5.63 mA/W at V=−1.48 V. The noise current spectrum shown in Figure 6E exhibits a minimum around zero bias, where the dark current is minimal and the differential resistance becomes maximal. The net photocurrent (Figure 6F) reaches a maximum value of 25.3 μA at V=−1.48 V, while a measurable photocurrent of 0.125 μA is still observed under zero-bias conditions. Finally, the minimum noise equivalent power (NEP) shown in Figure 6G is $2.22\times 10^{-9}$ W·H$z^{-1/2}$, whereas the maximum specific detectivity displayed in Figure 6H reaches $1.14\times 10^{8}$ cm H$z^{1/2}$ $W^{-1}$.

The incorporation of bR into the PEDOT:PSS matrix introduces a predictable physical trade-off between absolute electronic transport magnitudes and bio-inspired functionality. The decrease in peak responsivity and specific detectivity compared to pristine PEDOT:PSS controls primarily stems from the bulky, electrically insulating nature of the lipid–protein purple membranes [79,80]. We hypothesize that the spatial distribution of these biological patches partially disrupts the long-range electronic percolation networks of the conducting polymer, thereby increasing local transport resistance and lowering the overall absolute current levels. However, the fundamental advantage of integrating bR is not to enhance raw electronic conductivity, but to act as a primary photoactive sensitizer, imparting genuine light-sensitivity to the organic matrix. While pristine PEDOT:PSS exhibits excellent conductivity, it generally lacks an intrinsic, highly specific photoactive mechanism under zero-bias conditions. In contrast, the unidirectional light-induced proton pumping cycle of bR establishes a functional bio-interface capable of spontaneous charge separation. We propose that this mechanism enables the biohybrid device to generate the distinct, self-powered photocurrent observed at 0 V without requiring an external voltage supply. Furthermore, control devices comprising the PEDOT/buffer matrix without the protein (Phase 2) failed to display this zero-bias transient profile, and pristine bR-only films are known to exhibit near-insulating behaviors with current values restricted to the noise floor [79]. Consequently, we posit that a co-deposited architecture is strictly necessary: the bR actively sensitizes the system to light, while the PEDOT:PSS provides the essential electronic readout network. Together, this hypothesized interplay successfully bridges biological protonic activities with organic electronics, introducing biological-like temporal dynamics and synaptic memory capabilities completely absent in conventional organic photodetectors [80,81].

It is important to contextualize that the extended recovery time of approximately 200 s appears to be intrinsically linked to the biohybrid nature of the active layer. Unlike purely electronic, commercial silicon photodetectors that rely on the instantaneous drift of electrons and holes, we hypothesize that the transient photoresponse in the bR/PEDOT:PSS system may be governed by a combination of slower, biologically relevant mechanisms. First, the interplay between the light-induced proton-pumping cycle of bR and potential ionic mass transport within the hydrated organic matrix likely operates on extended temporal scales. Second, it is proposed that the structural complexity of the protein–polymer–electrode interfaces introduces localized trap states; the slow de-trapping of accumulated charges would yield a persistent photoconductivity (PPC) effect, manifesting as a prolonged baseline recovery tail [82]. However, rather than presenting a limitation, this slow, cumulative signal integration is a highly sought-after feature in bio-inspired optoelectronics. While traditional silicon detectors are fundamentally limited in biological settings due to their rigidity, lack of biocompatibility, and purely electronic nature [83], the unique ionic–electronic coupling and temporal memory (persistent photoresponse) of our device make it exceptionally well-suited for emerging applications. These encompass biocompatible neuromorphic engineering, artificial visual synapses, and soft wearable bio-interfaces, where biological-like signal retention is prioritized over absolute processing speed [81,83].

### 3.4. Comparative Analysis of Optimized Devices

The three optimized devices representative of each experimental phase—OPD_1.2 V, OPD_60%, and bR_1.2V_60%—were comparatively evaluated over the full bias range (±1.5 V) to assess the cumulative effect of each successive modification of the active layer. The key figures of merit are summarized in Table 4.

The J–V curves under dark and illuminated conditions reveal a clear progression in the electrical behavior of the three devices. OPD_1.2 V and OPD_60% exhibit similar current levels and ohmic behavior, confirming that the incorporation of 40% buffer solution does not significantly alter the charge transport properties of the PEDOT:PSS film. In contrast, bR_1.2V_60% shows a substantially lower dark current across the entire bias range, which we attribute to the lower effective conductivity of the hybrid film after incorporation of bacteriorhodopsin into the polymer matrix. These results demonstrate that the progressive incorporation of buffer solution and bacteriorhodopsin into the PEDOT:PSS active layer produces a systematic trade-off between electrical performance and biohybrid functionality. The buffer solution preserves the optoelectronic properties of the optimized PEDOT:PSS film while providing the ionic environment required for protein stabilization. The subsequent incorporation of bR modifies the absolute detection figures of merit but introduces a bioactive photoresponsive component whose contribution is most evident under zero-bias operating conditions.

### 3.5. Morphological Characterization by Field Emission Scanning Electron Microscopy (FESEM)

**Figure 7:**
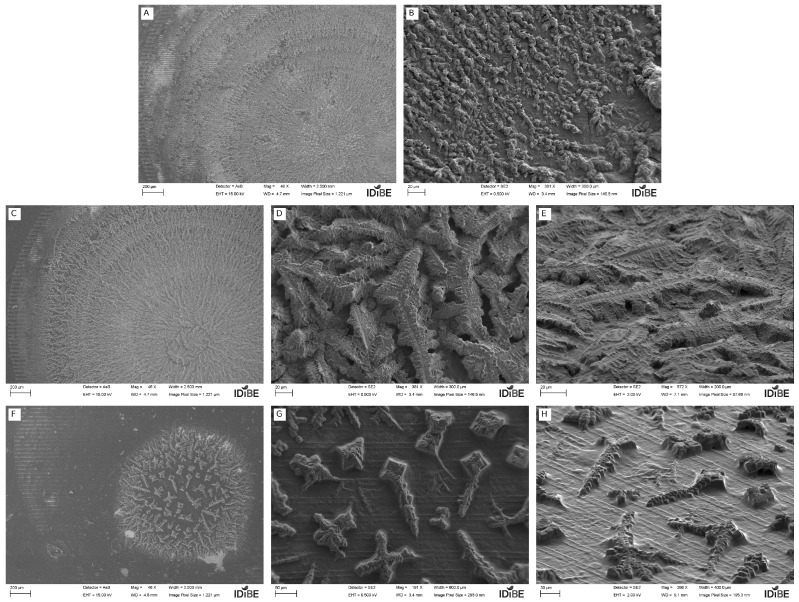
FESEM characterization of bacteriorhodopsin-based films at different magnifications. (**A**,**B**) bR_0.0V film showing irregular surface coverage and granular microstructure at low and intermediate magnification, respectively. (**C**–**E**) bR_1.2V film deposited under electric-field-assisted drying conditions, exhibiting more continuous surface coverage together with elongated and layered morphological features at increasing magnifications. (**F**–**H**) Hybrid bR_1.2V_60% film displaying localized crystalline inclusions and heterogeneous surface organization at increasing magnifications. Scale bars: (**A**,**C**,**F**) 200 μm; (**B**,**D**,**E**) 20 μm; (**G**) 50 μm; and (**H**) 30 μm.

The FESEM images of the bR_0.0V film (Figure 7A,B) reveal irregular and heterogeneous coverage of the interdigitated electrode surface, with aggregated domains of variable size distributed without apparent spatial order. At higher magnification (Figure 7B), the film exhibits a granular morphology composed of rounded aggregates approximately 1–3 μm in size, interconnected in short chain-like structures without preferential orientation.

In contrast, the bR_1.2V film (Figure 7C–E) presents more continuous and compact surface coverage after electric-field-assisted deposition. At intermediate magnification (Figure 7D), the aggregates appear more elongated and exhibit a more directional spatial distribution. While this macroscopic morphological arrangement suggests that the applied electric field influenced the deposition process, these surface-sensitive qualitative images should not be overinterpreted as direct proof of true electric-field-induced molecular-level organization. The tilted FESEM image shown in Figure 7E further reveals a layered morphology with visible thickness development above the substrate surface, where lamellar structures exhibit a directional arrangement consistent across the deposited region.

In the hybrid bR_1.2V_60% film (Figure 7F–H), the morphology changes significantly, showing a compact localized deposit together with large faceted crystalline structures displaying square and rectangular geometries, likely associated with buffer salt crystallization during drying. The presence of these residual salt aggregates might exert an influence on both the charge transport and the operational stability of the device. Regarding charge transport, it is hypothesized that these crystalline domains could act as localized electronic scattering centers and insulating structural defects within the organic matrix, potentially introducing carrier trapping sites. Despite the presence of these crystalline inclusions, the underlying PEDOT:PSS matrix remains visible as a continuous coating over the electrode fingers, which may explain the residual electrical conductivity and photoelectric response observed in the hybrid device. Furthermore, the potential hygroscopic nature of these residual salts might pose a challenge for long-term device stability. Preliminary evaluations of the biohybrid films over a 24-h period under ambient conditions suggested a noticeable increase in the dark current baseline and a shift towards a more linear electrical transport profile. This behavior could be attributed to the residual salts absorbing ambient moisture over time, leading to partial dissociation and the creation of localized ionic pathways. Such pathways would effectively lower contact barriers and increase overall film conductance, thereby elevating the dark noise floor of the detector. While an exhaustive and systematic study would be required to fully elucidate this degradation mechanism, these preliminary observations strongly suggest that optimizing post-deposition buffer removal could be a critical step toward preserving the morphological homogeneity and the long-term optoelectronic stability of these biohybrid sensors.

### 3.6. Dynamic Photoresponse of the Hybrid bR_1.2V_60% Photodetector

**Figure 8:**
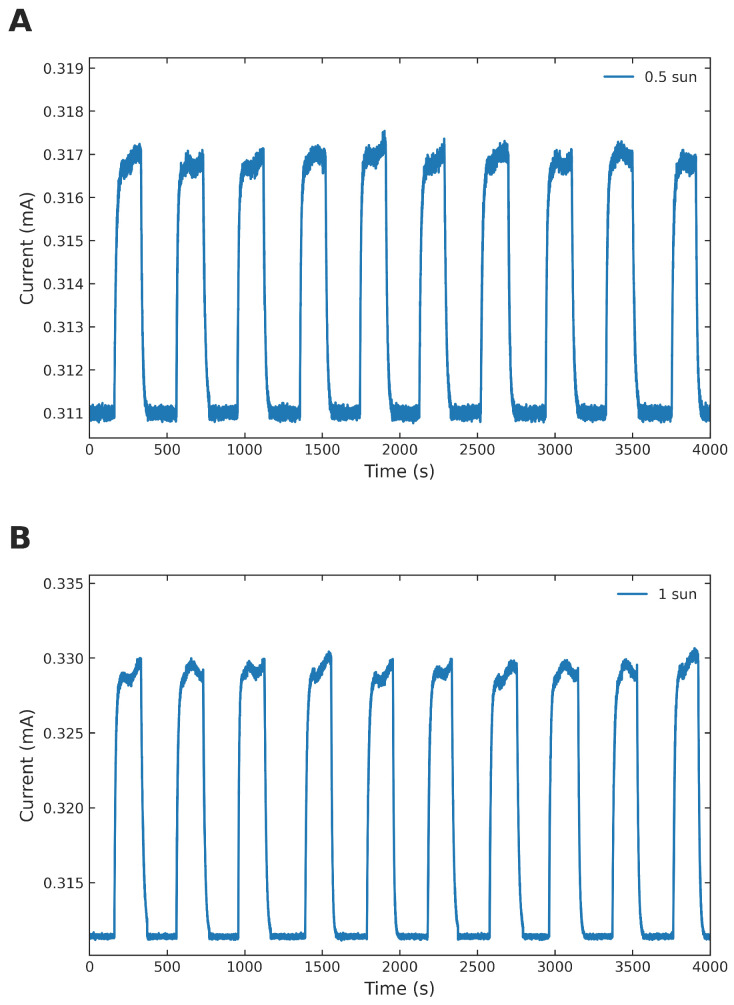
Dynamic photoresponse of the bR_1.2V_60% hybrid photodetector under ten consecutive ON/OFF illumination cycles at (**A**) 0.5 sun and (**B**) 1 sun.

The dynamic photoresponse of the bR_1.2V_60% hybrid photodetector was evaluated under periodic light modulation by recording the photocurrent as a function of time over ten consecutive ON/OFF cycles at two illumination intensities (0.5 and 1 sun). The results are summarized in Table 5 and Figure 8.

In dark conditions, the device maintains a stable baseline current of approximately 311 μA throughout the entire measurement, confirming the electrical stability of the hybrid film under ambient conditions. Upon periodic illumination, the photocurrent increases reproducibly over ten consecutive ON/OFF cycles for both illumination intensities. At 0.5 sun, the device reaches a peak ON current of (317.11±0.02) μA, corresponding to a photocurrent increment of $\Delta I_{\mathrm{ON}}=\left(6.19\pm 0.02\right)$ μA. Increasing the illumination intensity from 0.5 to 1 sun enhances the peak ON current to (329.68±0.07) l μA, yielding a photocurrent increment of $\Delta I_{\mathrm{ON}}=\left(18.54\pm 0.17\right)$ μA, corresponding to an approximately threefold increase in the net photocurrent compared with 0.5 sun (n=10 illumination cycles). Notably, both illumination intensities exhibit a similar transient response. The rise profile shows an initial rapid increase followed by a more gradual approach to the peak value, suggesting a dual-kinetic transient behavior often characteristic of organic mixed ionic–electronic systems [50]. Rather than signifying distinct and completely decoupled molecular pathways, we propose a theoretical framework in which this two-stage profile is governed by the asymmetric transport timescales of electronic and ionic carriers. The fast initial photocurrent surge is hypothesized to represent the near-instantaneous generation and drift of electronic carriers through the conjugated PEDOT chains. In contrast, the subsequent slower evolution toward saturation likely corresponds to the diffusion-limited migration of ions and protons within the hydrated matrix as they rearrange to balance the photoinduced electrostatic potential. Consequently, this transient signature is interpreted as a unified electrochemical relaxation process characteristic of biohybrid interfaces. Further dedicated transient studies would be required to unambiguously decouple these individual contributions. Upon switching off the illumination, the photocurrent progressively returns to the dark baseline during each OFF interval, exhibiting complete baseline recovery before the subsequent illumination cycle. The ten consecutive ON/OFF cycles recorded at both 0.5 and 1 sun show excellent reproducibility, with negligible cycle-to-cycle variation and low standard errors, confirming the stability, reversibility, and repeatability of the hybrid photodetector under periodic illumination. The approximately threefold increase in the net photocurrent between 0.5 and 1 sun, together with the excellent cycle-to-cycle reproducibility, demonstrates the capability of the hybrid device to provide stable and repeatable photoelectric operation under different illumination conditions.

### 3.7. Comparison with the State of the Art

The optoelectronic performance of the bR_1.2V_60% hybrid device can be contextualized within the broader landscape of bacteriorhodopsin-based photodetectors and biohybrid optoelectronic systems reported in the literature. Most previously reported bR photodetectors rely on sandwich-type architectures in which oriented bacteriorhodopsin films are deposited between transparent conductive oxide electrodes, typically ITO or FTO [79]. In these systems, device characterization is commonly focused on transient photocurrent generation, photovoltage response, or proton-coupled charge transport phenomena rather than on conventional photodetector figures of merit such as responsivity, noise equivalent power, or specific detectivity. Within this context, the hybrid device developed in the present work differs from most reported bR-based systems in several relevant aspects. First, the device employs a planar interdigitated electrode geometry instead of a vertical sandwich configuration, enabling lateral charge transport through the hybrid active layer. Second, bacteriorhodopsin is incorporated within a conductive PEDOT:PSS matrix rather than deposited as an isolated protein film, allowing simultaneous preservation of photoactivity and improved electrical conduction pathways. Similar hybridization strategies between bR and conductive or semiconducting materials have previously demonstrated enhanced interfacial charge-transfer behavior and improved photoelectronic coupling in biohybrid systems [80]. Although the absolute figures of merit obtained in the present work remain below those of conventional inorganic or fully synthetic organic photodetectors, the device exhibits consistent and reversible photoresponse over the measured cycles under ambient conditions together with measurable operation at zero bias. Furthermore, the methodology presented here provides a systematic evaluation framework for bacteriorhodopsin-based hybrid photodetectors using standard optoelectronic figures of merit, helping to bridge the gap between biological photoresponse studies and the characterization protocols commonly employed in the organic photodetector field.

## 4. Conclusions

In this work, a systematic study on the fabrication and optoelectronic characterization of biohybrid photodetectors based on bacteriorhodopsin and PEDOT:PSS deposited on interdigitated electrodes has been presented. The experimental strategy was organized in three sequential phases aimed at independently evaluating and refining the electric-field-assisted deposition conditions, the PEDOT:PSS/buffer composition, and the subsequent incorporation of bacteriorhodopsin into the active layer.

The results demonstrate that the application of an electric field during film drying significantly influences the optoelectronic performance of PEDOT:PSS films, with a drying voltage of 1.2 V yielding the best overall figures of merit among the tested values. The incorporation of a 40% buffer fraction into the PEDOT:PSS matrix preserves the photoelectric properties of the conductive polymer while providing the physicochemical conditions required for bacteriorhodopsin stabilization. Under these most favorable experimental conditions, the resulting hybrid bR_1.2V_60% device exhibited a consistent and reversible short-term photoresponse over the measured cycles under both static and dynamic illumination measurements, together with measurable operation under zero-bias conditions.

The hybrid device showed lower absolute figures of merit than the best-performing PEDOT:PSS reference devices, reflecting the reduced effective conductivity associated with incorporation of the biological component into the conductive matrix. Nevertheless, the device maintained reproducible photocurrent generation, low dark current, and reversible switching behavior during consecutive illumination cycles. FESEM characterization revealed substantial macroscopic morphological differences between films prepared with and without electric-field-assisted deposition, as well as the presence of crystalline inclusions associated with buffer salt precipitation in the hybrid films.

Overall, the results demonstrate that the combined use of electric-field-assisted deposition and conductive polymer/bacteriorhodopsin hybridization constitutes a viable strategy for the fabrication of biohybrid photoresponsive devices. The systematic formulation and evaluation approach presented here may also be extended to the integration of other photoactive biological materials into solution-processed optoelectronic platforms.

## Figures and Tables

**Table 1:** Optimal performance values in the polarization range (±1.5 V). Values are reported as mean ± standard error (n=5).

Device	$R_{\max}$ (mA/W)	${\mathrm{NEP}}_{\min}$ (W·H$z^{-1/2}$)	$D_{\max}^{*}$ (cm H$z^{1/2}$ $W^{-1}$)
OPD_0.0 V	1.72±0.03	$\left(1.74\pm 0.03\right)\times 10^{-9}$	$\left(1.22\pm 0.02\right)\times 10^{8}$
OPD_0.6 V	14.82±0.54	$\left(1.20\pm 0.02\right)\times 10^{-9}$	$\left(1.77\pm 0.03\right)\times 10^{8}$
OPD_1.2 V	35.65±1.36	$\left(5.99\pm 0.13\right)\times 10^{-10}$	$\left(3.55\pm 0.07\right)\times 10^{8}$
OPD_1.8 V	4.32±0.03	$\left(2.85\pm 0.01\right)\times 10^{-9}$	$\left(7.43\pm 0.04\right)\times 10^{7}$

**Table 2:** Volumetric proportions of PEDOT:PSS and buffer solution used for the fabrication of the control devices dried at 1.2 V.

Device	PEDOT:PSS (%)	Buffer Solution (%)
OPD_20%	20	80
OPD_40%	40	60
OPD_60%	60	40
OPD_80%	80	20

**Table 3:** Comparison of figures of merit for the buffer proportion series. Values are reported as mean ± standard error (n=5).

Device	$R_{\max}$ (mA/W)	${\mathrm{NEP}}_{\min}$ (W·H$z^{-1/2}$)	$D_{\max}^{*}$ (cm H$z^{1/2}$ $W^{-1}$)
OPD_20%	6.07±0.27	$\left(2.28\pm 0.27\right)\times 10^{-9}$	$\left(1.95\pm 0.78\right)\times 10^{8}$
OPD_40%	19.01±0.88	$\left(1.45\pm 0.25\right)\times 10^{-9}$	$\left(2.57\pm 1.28\right)\times 10^{8}$
OPD_60%	52.78±1.30	$\left(4.70\pm 1.02\right)\times 10^{-10}$	$\left(5.82\pm 1.52\right)\times 10^{8}$
OPD_80%	1.81±1.04	$\left(7.34\pm 0.11\right)\times 10^{-9}$	$\left(7.08\pm 4.09\right)\times 10^{7}$

**Table 4:** Comparative figures of merit of the optimized devices from each experimental phase, evaluated under optimal conditions within the ±1.5 V bias range. Values are reported as mean ± standard error (*n* = 5 for PEDOT:PSS devices and *n* = 3 for the hybrid bacteriorhodopsin device).

Device	$R_{\max}$ (mA/W)	${\mathrm{NEP}}_{\min}$ (W·H$z^{-1/2}$)	$D_{\max}^{*}$ (cm H$z^{1/2}$ $W^{-1}$)
OPD_1.2 V	(35.65±1.36)	$\left(5.99\pm 0.13\right)\times 10^{-10}$	$\left(3.55\pm 0.07\right)\times 10^{8}$
OPD_60%	(52.78±1.30)	$\left(4.70\pm 1.02\right)\times 10^{-10}$	$\left(5.82\pm 1.52\right)\times 10^{8}$
bR_1.2V_60%	(5.63±0.76)	$\left(2.22\pm 0.34\right)\times 10^{-9}$	$\left(1.14\pm 0.18\right)\times 10^{8}$

**Table 5:** Dynamic photoresponse parameters of the bR_1.2V_60% hybrid photodetector under periodic ON/OFF illumination. Peak ON current and $\Delta I_{\mathrm{ON}}$ are reported as mean ± standard error (n=10 illumination cycles).

Parameter	0.5 sun	1 sun
Dark baseline current (μA)	311.00	311.40
Peak ON current (μA)	(317.11±0.02)	(329.68±0.07)
$\Delta I_{\mathrm{ON}}$ (μA)	(6.19±0.02)	(18.54±0.17)
$t_{\mathrm{rise}}$ (10%→90%) (s)	∼30	∼24
Number of ON/OFF cycles	10	10
Illumination ON time per cycle (s)	180	180
Dark interval per cycle (s)	180	180

## Data Availability

The original contributions presented in this study are included in the article.
